# Predictability in a highly stochastic system: final size of measles epidemics in small populations

**DOI:** 10.1098/rsif.2014.1125

**Published:** 2015-01-06

**Authors:** Q. Caudron, A. S. Mahmud, C. J. E. Metcalf, M. Gottfreðsson, C. Viboud, A. D. Cliff, B. T. Grenfell

**Affiliations:** 1 Department of Ecology and Evolutionary Biology, Princeton University, Princeton, NJ, USA; 2 Office of Population Research, Woodrow Wilson School of Public and International Affairs, Princeton University, Princeton, NJ, USA; 3 Fogarty International Center, National Institutes of Health, Bethesda, MD, USA; 4 Department of Medicine, Landspítali University Hospital, Reykjavík, Iceland; 5 Faculty of Medicine, School of Health Sciences, University of Iceland, Reykjavík, Iceland; 6 Department of Geography, University of Cambridge, Cambridge, UK

**Keywords:** measles, dynamics, epidemiology, small populations

## Abstract

A standard assumption in the modelling of epidemic dynamics is that the population of interest is well mixed, and that no clusters of metapopulations exist. The well-known and oft-used SIR model, arguably the most important compartmental model in theoretical epidemiology, assumes that the disease being modelled is strongly immunizing, directly transmitted and has a well-defined period of infection, in addition to these population mixing assumptions. Childhood infections, such as measles, are prime examples of diseases that fit the SIR-like mechanism. These infections have been well studied for many systems with large, well-mixed populations with endemic infection. Here, we consider a setting where populations are small and isolated. The dynamics of infection are driven by stochastic extinction–recolonization events, producing large, sudden and short-lived epidemics before rapidly dying out from a lack of susceptible hosts. Using a TSIR model, we fit prevaccination measles incidence and demographic data in Bornholm, the Faroe Islands and four districts of Iceland, between 1901 and 1965. The datasets for each of these countries suffer from different levels of data heterogeneity and sparsity. We explore the potential for prediction of this model: given historical incidence data and up-to-date demographic information, and knowing that a new epidemic has just begun, can we predict how large it will be? We show that, despite a lack of significant seasonality in the incidence of measles cases, and potentially severe heterogeneity at the population level, we are able to estimate the size of upcoming epidemics, conditioned on the first time step, to within reasonable confidence. Our results have potential implications for possible control measures for the early stages of new epidemics in small populations.

## 1. Introduction

Measles is a highly contagious and strongly immunizing infection of the respiratory system [1]. Owing to its high transmissibility and the lifelong immunity procured by infection, its epidemiology is conditional on the birth of susceptible individuals. As such, the temporal dynamics of measles are typically strongly oscillatory, driven seasonally by the increased contact rate among young children during school periods [2–4], assuming the population is large enough to sustain the infection. The critical community size, defined as the size of a population required to sustain the disease at an endemic level, is estimated to be between 250 000 and 500 000 [5–7]. In large populations, measles has been extensively studied, typically demonstrating biennial dynamics in developed countries prior to the introduction of vaccines [8,9]. These modelling efforts are typically based on a class of continuous-time systems of differential equations, such as the SIR and SEIR compartmental models. Mechanistically, these models provide a good description of the driving mechanisms behind infections such as measles, which have a well-defined infectious period, are directly transmitted and yield lifelong immunity to those who recover from the infection [1]. SIR-like models also assume, however, a certain level of homogeneous mixing between individuals in the population. In many large population studies, such as in [10], these assumptions hold reasonably well: the populations are large and spatially compact enough to guarantee sufficient mixing within the population and to ensure that the disease remains endemic.

In small populations, however, the dynamics of measles cases are different. Susceptible individuals accumulate when measles is absent; then, driven by stochastic recolonization, an epidemic may sweep through a large fraction of the susceptible population very quickly, only to go extinct abruptly as susceptible counts fall below the threshold required for endemicity. This results in very sharp, spiky epidemics, whose timing may be impossible to predict; they are described as Type III by Bartlett [5]. Methods typically used in the analysis of time-series or in dynamical systems theory are not adapted to the study of temporal changes of measles incidence in such small populations. Nonetheless, scaling analysis in small populations has revealed that some level of predictability can be found within the statistics of epidemic size and duration distributions, despite the small number of epidemics observed in the recorded data [11,12].

A discrete-time adaptation of SIR-like models was developed by Finkenstädt & Grenfell [13]. The TSIR model is a simple and computationally inexpensive system of difference equations, which can be parametrized against observed incidence time-series and birth data, and is able to estimate non-analytical, time-varying contact and transmission rates. It has been successfully used in the analysis of seasonal variation of measles in several systems with large populations [14,15]. In addition, the model has been applied to small populations that demonstrate persistent, periodic dynamics due to strong coupling with nearby large populations [10,16].

However, little has been done on applying the TSIR model to subendemic populations with recurring and episodic outbreaks. Datasets on the incidence of diseases such as influenza and measles have been created from medical and parish records in small and isolated populations, where the disease dynamics are dominated by the stochastic importation of infected individuals. These datasets have been studied from the perspective of historical geography, where the occurrence and spatio-temporal spread of epidemics are explained by features of the landscape and of local populations [17,18]. Despite the availability of these datasets, however, no inference methods have yet been applied to the problem of characterizing the dynamics of disease spread in these unique systems.

In this paper, we address the question of predictability of measles epidemics in subendemic, isolated populations. First, we present data on the demographics and disease incidence in prevaccination-era Bornholm, the Faroe Islands and four districts in Iceland. Then, we summarize the TSIR model and fit the parameters of the model to the data. After generating predictions for the evolution of each epidemic, we compare the mean predictions with the original time-series, and the predicted size of each observed epidemic. Finally, we discuss factors which may influence the accuracy of predictions, and possible improvements to the data and methods used for improved results.

## 2. Material and methods

### 2.1. Data

Measles incidence data were obtained for Iceland, from 1901 to 1965, from [17]. This dataset consists of monthly figures for measles cases reported in 47 medical districts (*læknishérað*), originally sourced from *Heilbrigðisskýrslur* (Public Health in Iceland). Medical districts, the basic reporting unit for disease data in Iceland, are composed of *hreppar* (communes) that are roughly equivalent to English parishes or American townships. Major revisions to the boundaries of medical districts took place twice during the study period: in 1907 and 1932. Monthly incidence data for the Faroe Islands, from 1912 to 1965, were obtained from [18]; these data were originally sourced from [19]. For Bornholm, monthly measles incidence data from 1925 to 1965 were acquired from [20].

Demographic data for Iceland were obtained from Iceland, Statistics Iceland. www.statice.is. Annual data on population and number of live births for the entire country were taken from Iceland, Statistics Iceland: Births by months 1853–2012 www.statice.is/Statistics/Population/Births-and-deaths. Decennial population data from 1901 to 1965, for 262 municipalities, were obtained from Iceland, Statistics Iceland: Population by municipalities 1901–1990 www.statice.is/Statistics/Population/Municipalities. Municipal borders changed from three to five times during the study period, depending on the municipality. In addition, many municipalities had missing data. Medical districts and municipalities were matched based on names. Several matched districts were discarded either due to missing population data, or lack of confidence in the matching of the geographical boundaries. With the data available, we were able to match four district–municipality pairs: Akureyri, Reykjavík, Hafnarfjörður and Vestmannaeyjar. It is worth noting that matched medical district–municipality pairs may not encompass the exact same area, but one may be a (potentially partial) subset of the other.

Data on the demographics of the Faroe Islands were taken from the Statistical Yearbooks of Denmark published by Statistics Denmark (Denmark, Danmarks Statistik www.statistikbanken.dk) and from Statistics Faroe Islands (Hagstova Føroya www.hagstova.fo). Annual data on population and births from 1901 to 1965 were found in aggregated form for all of the islands in the Faroe archipelago.

Demographic data for Bornholm were collected from several publications in Denmark, Danmarks Statistik www.statistikbanken.dk. Annual population data for Bornholm were obtained from Denmark, Danmarks Statistik: Population 1 January by islands www.statbank.dk/statbank5a/SelectVarVal/Define.asp?MainTable=BEF4&PLanguage=1, which contains detailed statistical information collected by Statistics Denmark. Pre-1930 annual birth data were obtained from the *Ægteskaber, Fødte og Døde* (Marriages, Births and Death) available from Denmark, Danmarks Statistik: Ægteskaber, Fødte og Døde www.dst.dk/pukora/epub/upload/20304/aefodo1921-1925.pdf www.dst.dk/pukora/epub/upload/20305/aefodo1926-1930.pdf). Post-1930 annual birth data were obtained from *Befolkningsudvikling og sundhedsforhold 1901–60* (Population, Development and Health 1901–1960), from Denmark, Danmarks Statistik: Befolkningsudvikling og sundhedsforhold www.dst.dk/pukora/epub/upload/19335/befsund.pdf.

Table 1 presents the mean populations and birth rates over the study period. The reported incidence and births for Bornholm, the Faroe Islands and four districts of Iceland, can be found in the electronic supplementary material. The data and code used in this paper can be found online (Complete dataset and code, Github repository http://github.com/QCaudron/SmallPopTSIR).

**Table 1.** Mean population sizes, birth rates and sensitivity thresholds *τ* for each locality. Population sizes and annual birth rates per thousand are given as the mean over the study period. Thresholds were fit by maximizing the correlation between the mean simulated epidemic time-series and the reported incidence data.

locality	population	birth rate	*τ*
Bornholm	47 100	19.4	15
Faroe Islands	28 200	29.4	15
Reykjavík	47 100	24.1	18
Hafnarfjörður	6000	22.4	8
Akureyri	7000	22.7	19
Vestmannaeyjar	3600	23.5	7

### 2.2. The TSIR model

The time-series SIR model [13] is a discrete-time, stochastic model of disease progression written in terms of a set of difference equations. Assuming that the infection is fully immunizing and that the infectious period is well-defined, then the evolution of the number of infected cases, *I_t_*, can be written as follows:2.1
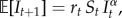
where *S_t_* is the number of susceptible individuals at time *t*, seasonal contact rates are represented by the parameter *r_t_*, where *r_t_* = *r_t_*_+_ *_P_* is periodic with period *P* time steps per year, 0 < *α* < 1 is a mixing parameter allowing for nonlinearities due to the model's discrete-time approximation and inhomogeneous population mixing, and where 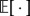 denotes the expectation operator. The time step is set as the generation time of the infection. Then, the number of susceptible individuals is defined by2.2
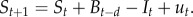
Here, *B_t_*_−*d*_ is the number of births that occurred *d* generations prior to *t*, where the delay *d* represents a period of protection from infection due to maternal immunity, set at four months [1]; and *u_t_* is additive noise with 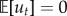. If the number of susceptible individuals *S_t_* fluctuates around a mean 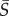 such that 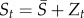, then, from equation (2.2), the dynamics of the susceptible individuals around their mean 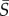 are given by2.3
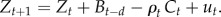
The observed number of cases, *C_t_*, is related to the inferred number of actual cases by *I_t_* = *ρ_t_C_t_*. In ideal populations, the observation scaling factor *ρ_t_* represents the reciprocal of the reporting rate, such that *ρ_t_* > 1 signifies an underreporting of cases by the factor 1/*ρ_t_*. When birth information comes from a different geographical region than that of the time-series of disease incidence, then *ρ_t_* is influenced by this confounding factor: *ρ_t_* then becomes both a reporting rate and a correcting factor for this geographical discrepancy.

As the TSIR model assumes that all individuals will eventually become infected, the incidence must therefore track the number of births. Successive iteration of equation (2.3) yields2.4
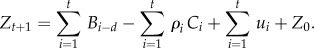
Assuming *u_t_* is small, *ρ_t_* can be estimated using local regression methods between the cumulative births and cumulative observed cases. Then, *Z_t_* can be found as the residuals of this regression.

### 2.3. Fitting

The time step in the difference equations (2.1) and (2.2) is fixed at the generation time of the infection. For measles, the period of time from infection to recovery is approximately two weeks [1]. Owing to the very spiky nature of the reported incidence data (whose derivatives are non-smooth due to low sampling rates), interpolation must be done such that peaks in the data are not missed or reduced. As such, a linear interpolant with an integer multiple of the number of points per year was used. This yielded 24 time points per year, thus maintaining the maximum values of the peaks in the data, and fixing the generation time at just over 15 days.

Populations and live births, assumed to be smooth, were interpolated cubically. There are large intervals between some of the reported demographics data; however, Finkenstädt & Grenfell [13] report that the regression for reconstructing susceptibles is robust to pronounced changes in birth rates.

The observation factor *ρ_t_* was estimated using Gaussian process regression, given the births and reported cases. Gaussian processes yield the best linear unbiased predictions of values in between observations, providing smooth regression curves that fit the data well. Finkenstädt & Grenfell [13] employed local regression methods and suggest that splines would also provide good results; our analysis shows that, for such stepwise data (due to the very discrete nature of sudden epidemics), Gaussian processes resulted in more robust fits. Once found, *ρ_t_* is the derivative of the Gaussian process regression for the cumulative number of births, with respect to the cumulative number of cases, and *Z_t_* are the residuals of the regression.

The mean number of susceptibles 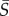 was estimated marginally by profiling the likelihood of the logarithmic form of equation (2.1),2.5
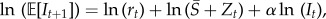
after which the seasonal contact rates *r_t_* were estimated conditionally on 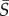. The mixing parameter was fixed at *α* = 0.97, as in [15], implying a small, nonlinear inhomogeneity, yet not significantly impacting transmission dynamics between large and small epidemics.

In summary, the TSIR model is fit entirely from a time-series of births, *B_t_* and of observed disease incidence, *C_t_*. The cumulative births, 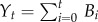, are regressed against the cumulative observed incidence, 
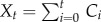
, to yield *ρ_t_* as the gradient of the regression curve, with the residuals of this regression giving the dynamics of the susceptible population *Z_t_* about their currently unknown mean, 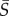. This regression takes the form *Y_t_* = *ρ_t_ X_t_* + *Z_t_*. The mean number of susceptible individuals, 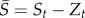 is estimated by profiling the likelihood of a log-form of equation (2.1) with respect to 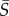. Then, fixing 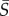 to maximize the likelihood, the coefficients of *r_t_* are fit by least squares.

### 2.4. Predictions

Using the TSIR model as defined by the system of equations (2.1) and (2.2), predictions for epidemic dynamics were made by sampling the incidence *I_t_*_+__1_ from a negative binomial distribution2.6
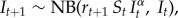
with mean 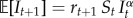 and shape parameter *k* = *I_t_*.

Owing to the abundance of zeroes in the incidence time-series, initial conditions cannot simply be taken as the point (*I*_0_, *S*_0_). Instead, each epidemic must be simulated independently, with initial conditions given by the data at the time that the epidemic begins. For each epidemic, we fix the initial number of infected cases and susceptible individuals as per the observed data and the reconstructed susceptible time-series, respectively, and allow the simulation to continue until the next epidemic begins. Thus, we always simulate the same number of epidemics as given by the incidence data, where each epidemic is simulated given only the data available at the onset of that epidemic.

In order to clearly establish the time of onset of an epidemic, a sensitivity threshold must be set. Let 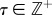 define the number of reported infected cases necessary for any particular biweek period to be considered part of an epidemic. In order for epidemic detection to be robust, we convolve the incidence time-series with a nine-point Hanning window and round to the nearest integer; then, any biweeks where the smoothed series is greater than *τ* are to be counted as part of an epidemic. This ensures that points slightly under *τ* are not penalized, should the next few points be greater than *τ*. This also reduces the risk of detecting sporadic recolonizations that fail to become full epidemics.

A choice of *τ* = 1 ensures that all available non-zero data are used. However, many potential epidemics go extinct before propagating through the population, especially in highly heterogeneous populations. As such, using *τ* = 1 would cause a number of strongly overestimated epidemics. We therefore treat τ as a sensitivity parameter, and fit it by selecting the sensitivity threshold which yields the highest correlation between the mean predicted epidemic traces and the incidence data, as defined by the coefficient of determination, *R*^2^. Then, the first point in a sequence of time steps defined by this method as belonging to an epidemic is considered the onset of that epidemic.

## 3. Results

### 3.1. Dynamics

After fitting parameters as described above, predicted epidemic time-series were generated for each of the six localities, using the sensitivity thresholds reported in table 1. The simulated time-series, computed from the difference equations (2.2), (2.4) and (2.5), are effectively *n*-step-ahead predictions, with *n* representing the duration of an epidemic.

**Figure 1.**
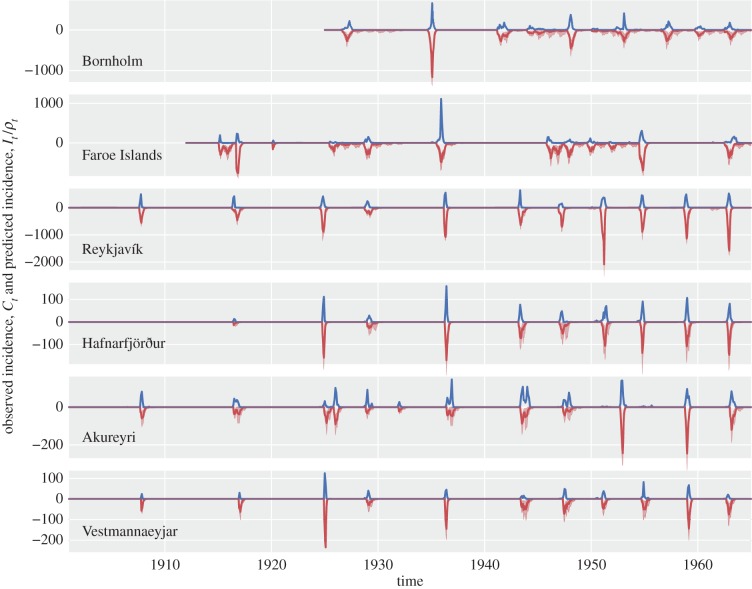
Reported and predicted biweekly incidence for Bornholm, the Faroe Islands and four localities in Iceland. The observed data are in blue. For the predicted time-series, the mean value of incidence simulations is plotted as a dark red line, with 95% CIs given in light red. Bornholm: *R*^2^ = 0.78; Faroe Islands: *R*^2^ = 0.55; Reykjavík: *R*^2^ = 0.73; Hafnarfjörður: *R*^2^ = 0.86; Akureyri: *R*^2^ = 0.80; Vestmannaeyjar: *R*^2^ = 0.77.

Figure 1 shows the time-series of the number of reported and predicted cases in all six localities. Predictions are plotted as the mean incidences across 10 000 simulations, with their respective 95% CIs. High temporal synchronicity can be seen in the Icelandic localities. In general, epidemics in Iceland are of shorter duration, while those in Bornholm and the Faroe Islands are not quite as spiky. In all localities, epidemics seem to occur more frequently in the latter half of the time-series, perhaps due to an increase in birth rates after the baby boom.

The reported coefficient of determination *R*^2^ has been corrected by removing points where both the observed and predicted time-series are simultaneously zero, to reduce inflation of the coefficient due to the large number of zeroes in the time-series. Overall, good agreement is generally found with the observed data, with the highest correlation being in Hafnarfjörður, a small district about 10 km from Reykjavík. The worst fit is found in the Faroe Islands, by a significant margin; predictions here are characterized by a number of failed extinctions, general overestimation of epidemic sizes and durations, except for the single, large observed epidemic, which is significantly underestimated. It has been suggested that these very large epidemics may have fundamentally different dynamics [12], which would cause difficulties in parameter inference.

A number of predicted epidemics have a right shoulder, where the model predicts that epidemics take longer to go extinct than those observed. Depending on locality, many of these shoulders are small (Akureyri, Hafnarfjörður and Vestmannaeyjar). For other localities, predicted epidemics may fail to go extinct entirely, demonstrating cyclical behaviour until the beginning of the next epidemic (Bornholm, the Faroe Islands and Reykjavík). This may indicate that populations are strongly heterogeneous, and that the inhomogeneity parameter, fixed at *α* = 0.97 for these simulations, is an overestimate.

**Figure 2.**
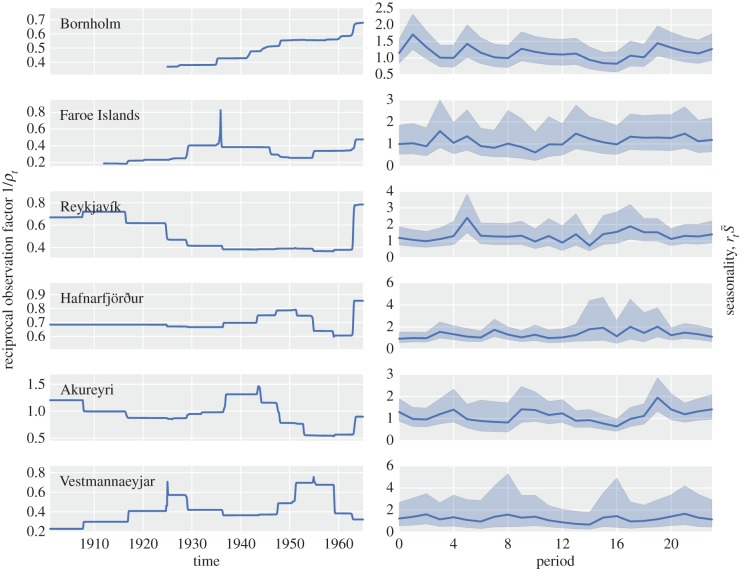
Observation factors and seasonalities. Seasonality is plotted as a function of the biweek, with 95% CIs in light blue.

Inferred observation scaling factors and seasonal transmission rates are shown in figure 2. The inferred seasonalities have wide distributions, demonstrated by their large confidence intervals. This can be explained by the highly stochastic nature of measles recolonizations into their respective localities, which is the primary driver for when epidemics occur. This is in contrast to the seasonality inferred in studies of large populations, such as that of England and Wales in [13], where significant seasonal trends were found, and matched well with school-based contact times. When the transmission rates were fixed to a constant, such that 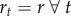, neither the inferred parameters nor the predicted dynamics changed significantly.

### 3.2. Predictability in epidemic sizes

**Figure 3.**
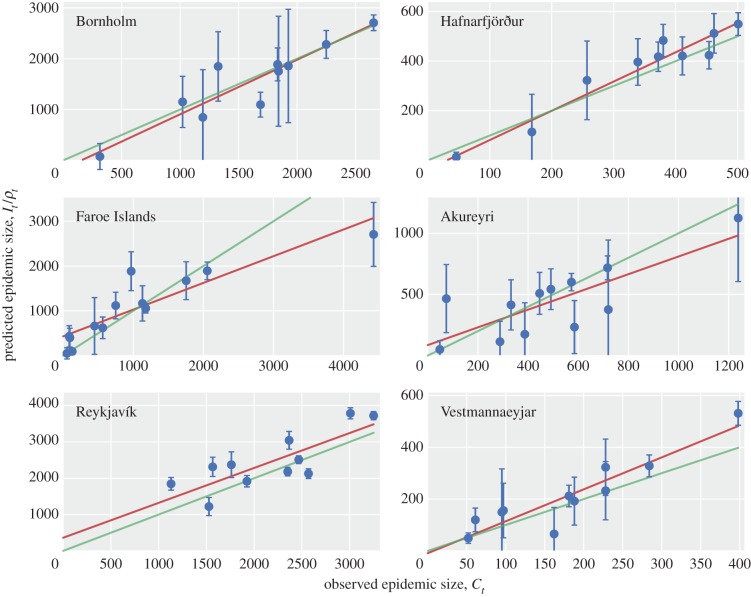
Predictability of epidemic sizes. The mean predicted size of each epidemic as a function of its observed size, from 10 000 simulations. Red lines are the regression lines with the follow coefficients of determination and slopes—Bornholm: *R*^2^ = 0.76, gradient = 1.07; Faroe Islands: *R*^2^ = 0.77, gradient = 0.60; Reykjavík: *R*^2^ = 0.64, gradient = 0.96; Hafnarfjörður: *R*^2^ = 0.88, gradient = 1.18; Akureyri: *R*^2^ = 0.49, gradient = 0.72; Vestmannaeyjar: *R*^2^ = 0.76, gradient = 1.23. The green line is the zero-intercept, gradient-one line representing a one-to-one match between observation and prediction.

Rather than considering a point-wise comparison between the predicted and observed epidemic time-series, a potentially more robust measure of predictability is the total number of infected cases that a particular epidemic will generate. We define the size of an epidemic as the sum of reported cases *C_t_* for observed data, or *I_t_*/*ρ_t_* for predicted data, from the first time point in an epidemic to the time point before the next epidemic begins. Figure 3 shows the mean predicted epidemic size for each observed epidemic for the six localities. Several of these localities show a strong linear relationship, with near-zero intercepts and gradients around one. Again, the highest correlation between predicted and observed epidemic sizes is found in Hafnarfjörður, with a coefficient of determination of *R*^2^ = 0.88.

## 4. Discussion

Predictions of epidemic sizes can be made with a significant level of certainty, despite sparse demographic data for all localities, mismatching incidence and demography information in Iceland, and strong spatial barriers to population mixing in the Faroe Islands. Hafnarfjörður shows the best correlation between predicted and observed epidemic sizes, potentially due to its geography—it is a community just outside the capital city of Reykjavík, small enough that district and municipality borders may match well. Perhaps for similar reasons of matching data streams and no major geographical restrictions to population mixing, Bornholm and Vestmannaeyjar also show good correlations between expected and observed epidemic sizes. Epidemic sizes in both Akureyri and the Faroe Islands, however, are underestimated with respect to the observed epidemic sizes. For Akureyri, this could be due to an underestimation in the actual number of births in the area, caused by having a smaller municipality than the related medical district. This would generate a smaller reconstructed susceptible pool, reducing the sizes of predicted epidemics. For the Faroe Islands, the regression seems to be skewed to a smaller gradient by a single outlier whose size is grossly underestimated. Depending on the quality of the data, therefore, predictions about the size of a future epidemic can be made with some confidence.

Data streams that do not represent exactly the same physical space, measurement or system, are common problems in epidemiology and, indeed, in many fields where observation and data collection are non-trivial tasks, or where the system cannot be observed directly. Our results show the effects of data streams that do not quite match: whether due to data aggregation in the Faroe Islands, or mismatching (and changing) borders for incidence and demographics data in Iceland, model fitting can be made more difficult. As an extreme case where data are abundant, and where demographic information is representative of the same regions as those of the measles incidence time-series, the TSIR model fitting performed by Finkenstädt & Grenfell [13] on 60 cities in England and Wales was highly successful.

Many improvements could be made to the dataset used in this paper to improve predictability of epidemic sizes. An understanding of where both the medical and municipal borders lie would allow a much larger number of districts to be fit confidently; in addition, an underlying spatial model could be used to counter the border changes and to analyse the data for spatial correlations. Disaggregated incidence and birth information for the large islands in the Faroe archipelago could be used to consider the separate island populations, each of which would have higher internal mixing, with a lower inter-island homogeneity. Noting that, in the second half of the Icelandic time-series, epidemics were perhaps becoming more regular, it may also be valuable to model the interepidemic intervals for longer time-series.

With the current data, possible improvements include the use of statistical models such as trajectory matching or hidden Markov models to infer a biweekly incidence rate rather than using a linear interpolant, or the addition of an Exposed state variable to allow for exposed but not infectious individuals in a ‘TSEIR’ model.

Nonetheless, we have demonstrated that a strong signal of SIR-like epidemic dynamics can be found even in systems dominated by noisy importations. These well-known time-series [17,18] are a paradigm for epidemics in small populations. Their sporadic nature is caused by long periods of time between stochastic importations, followed by extinctions. As such, their dynamics are reminiscent of pandemics, where a significant proportion of the population is susceptible to infection at the onset of the epidemic. This interesting analogy extends to the lack of an observed seasonal signature. Our analysis reveals that, even from a highly stochastic incidence time-series and limited demographic data, reasonable predictions for the final size of an epidemic can be made, conditioned on the state of the system at epidemic onset. These results may have implications for the control of future epidemics, potentially informing response strategies based on the predicted size of an epidemic that was just initiated from a recolonization event.

Significant spatial restrictions to population mixing, such as the fragmented island geography of the Faroe archipelago, may impact the level of predictability that can be found in these results. On the whole, however, we find that a homogeneous mass-action assumption is fairly successful overall—with *α* ≈ 1, the model fits the data well for most localities. Indeed, Earn *et al.* [21] report that heterogeneous transmission is not required to obtain realistic dynamics in measles models. This is in contrast to the scaling analyses of Rhodes & Anderson [11,22], whose work suggests that heterogeneous dynamics are necessary to explain the distributions of final epidemic sizes and durations. Given the crudeness of the data, however, these results are tentative, and comparisons of our approaches to theirs are a fruitful area for future work.

## Supplementary Material

Observed Incidence

## Supplementary Material

Births
