# Supplementary figures and images for: Predictability in a highly stochastic system: final size of measles epidemics in small populations
Source: J R Soc Interface. 2015 Jan 6;12(102):20141125. doi: 10.1098/rsif.2014.1125 (PMC4277111; doi:10.1098/rsif.2014.1125)

Incidence per biweek

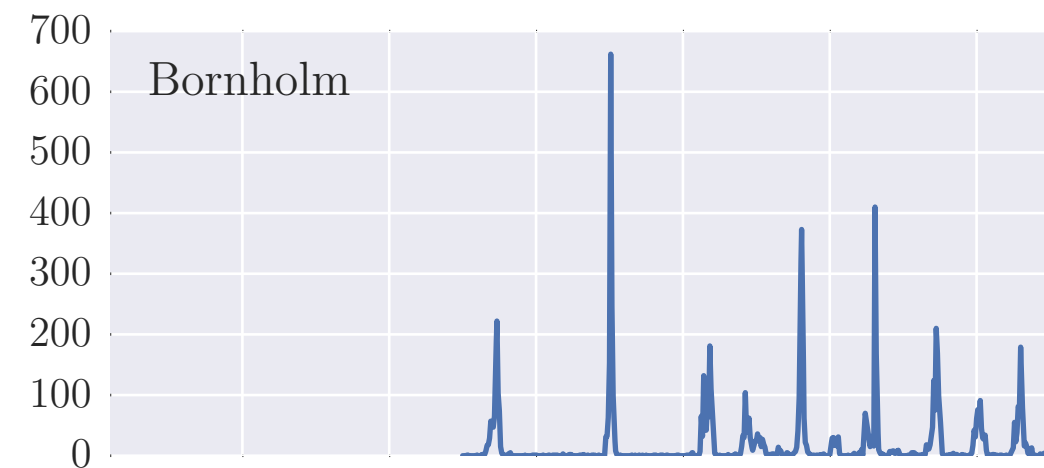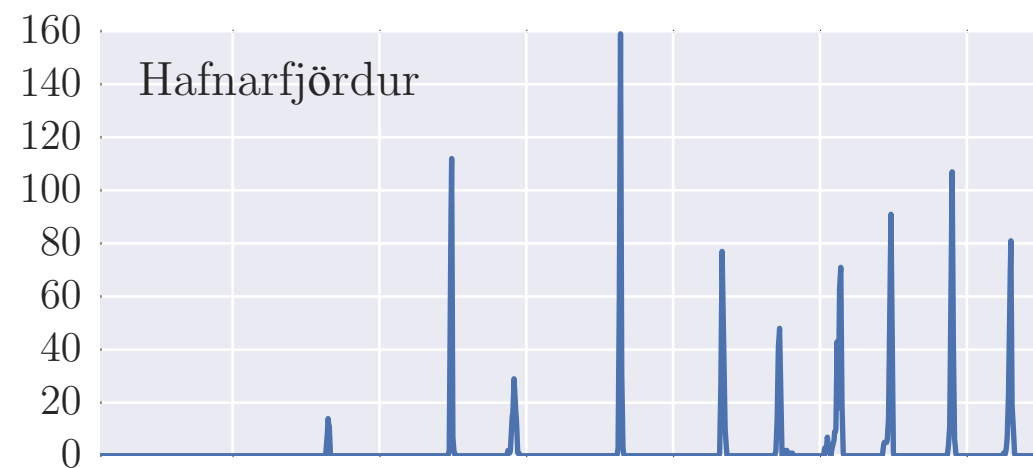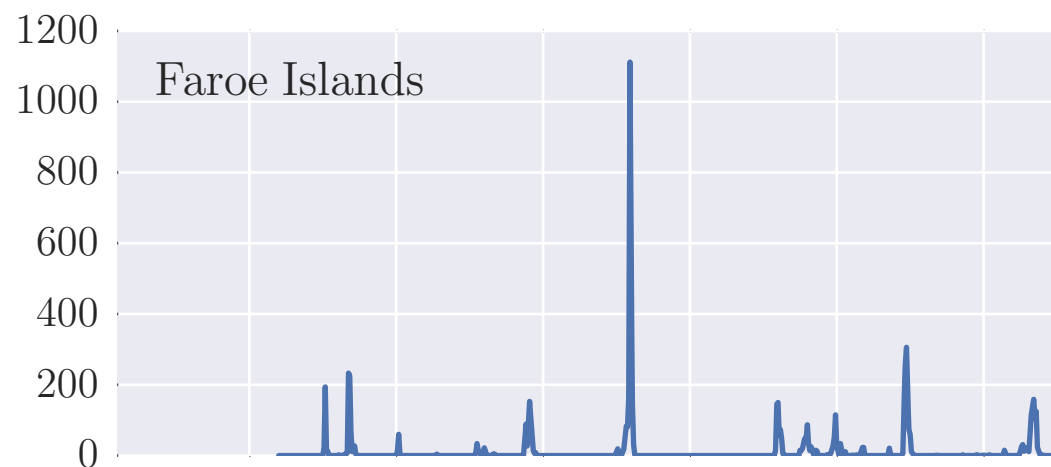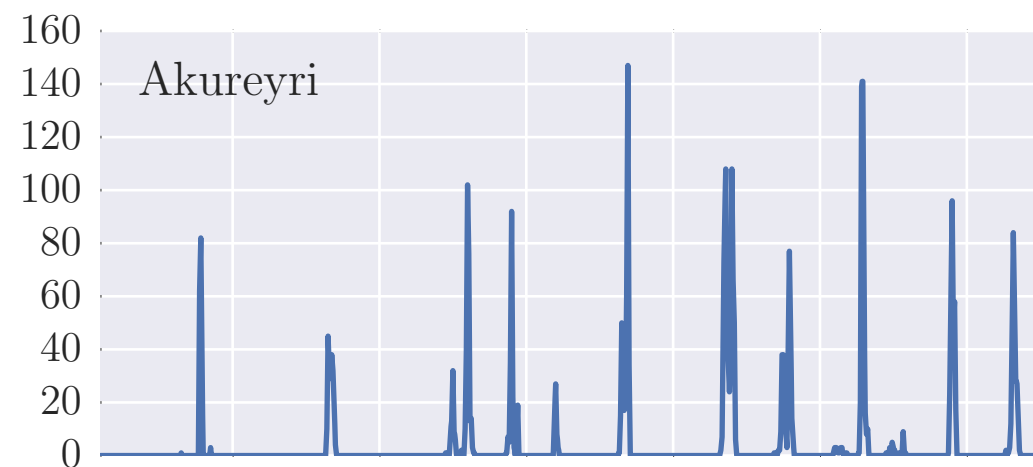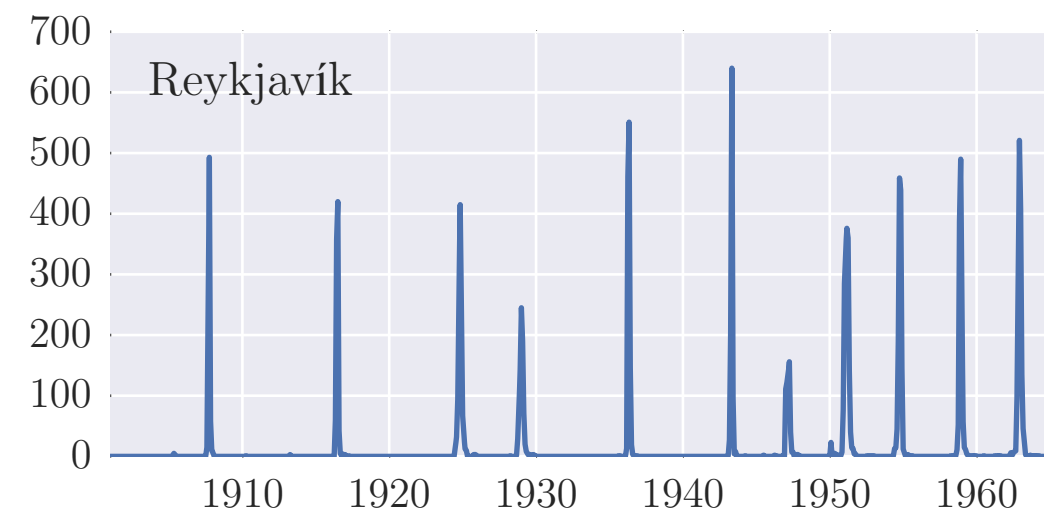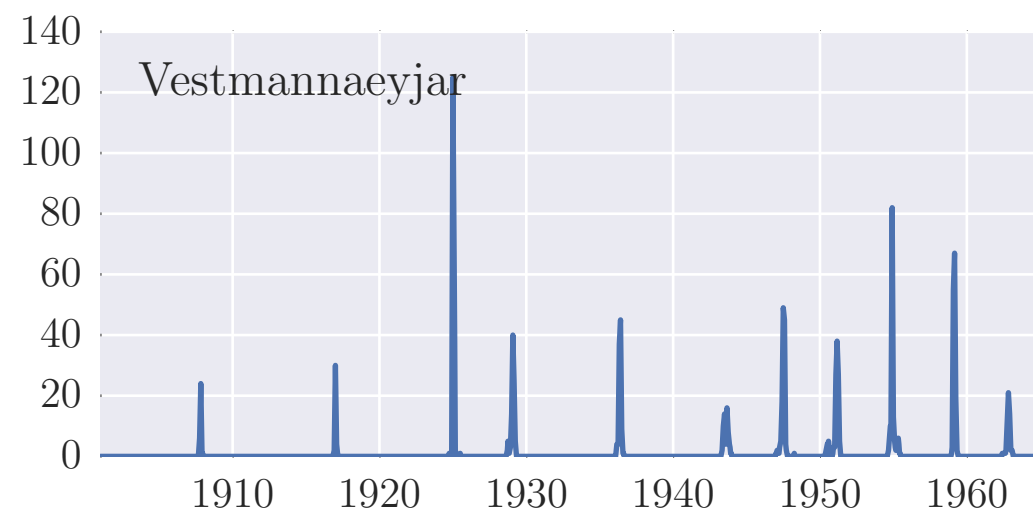

Supplement: Observed Incidence [file rsif20141125supp1.pdf]

Births per biweek

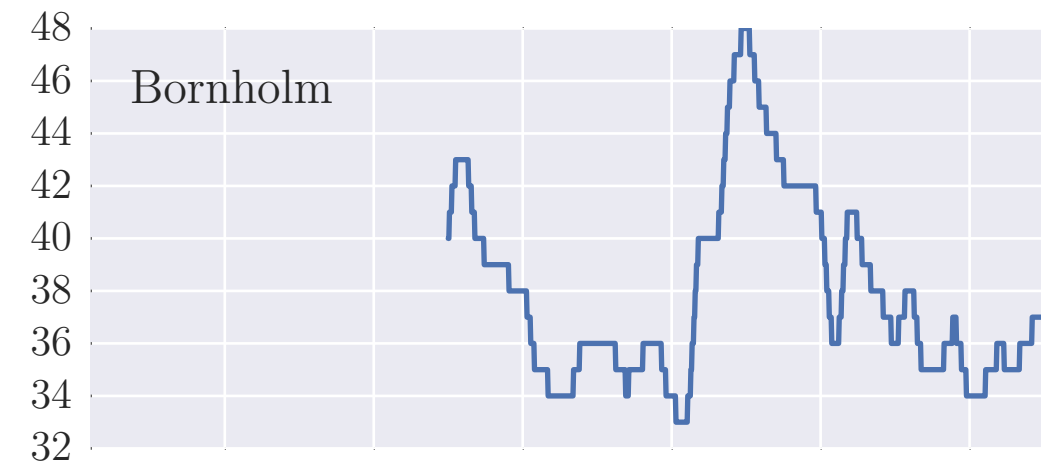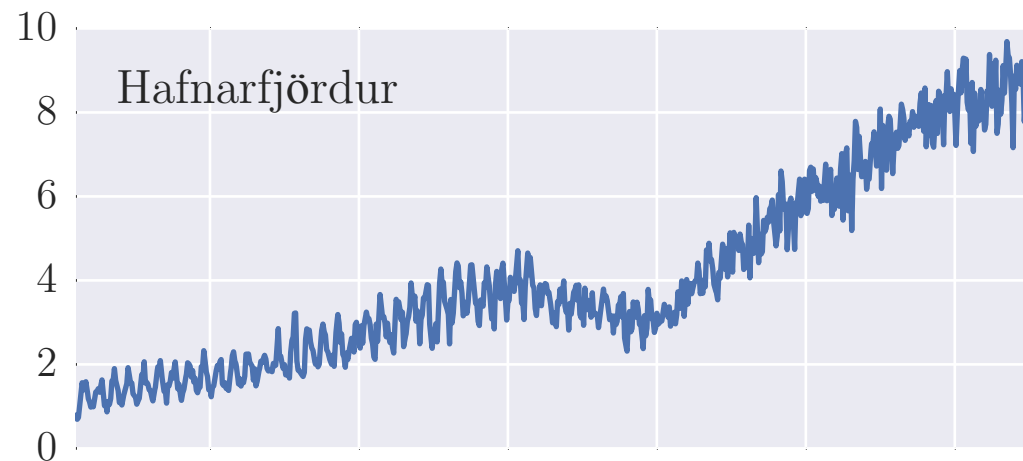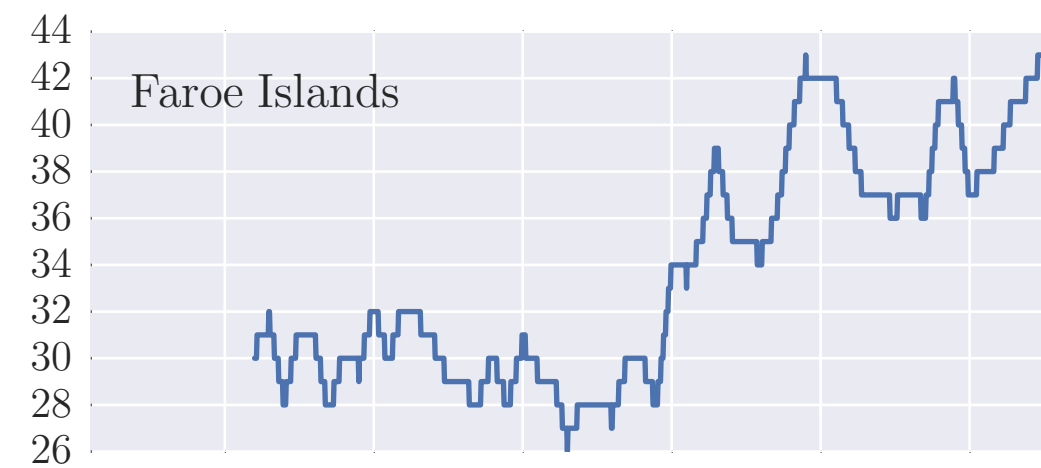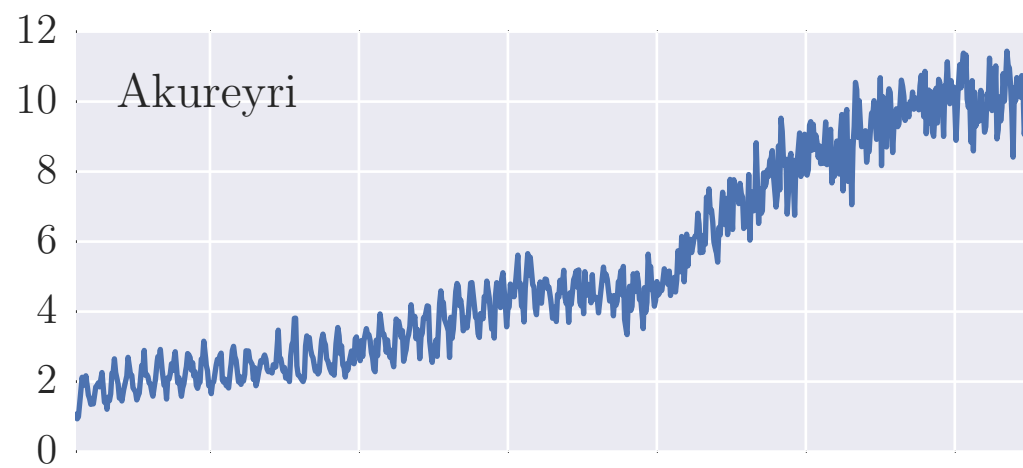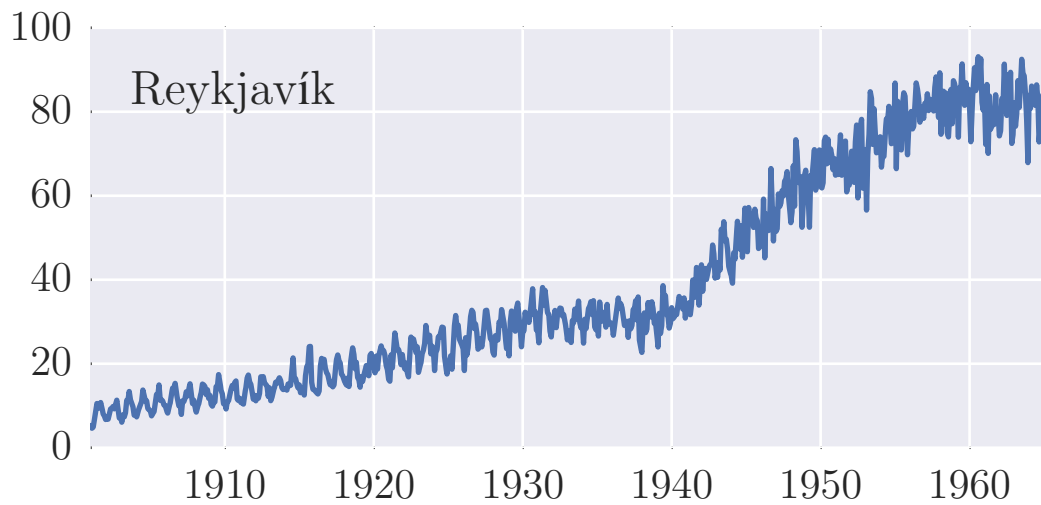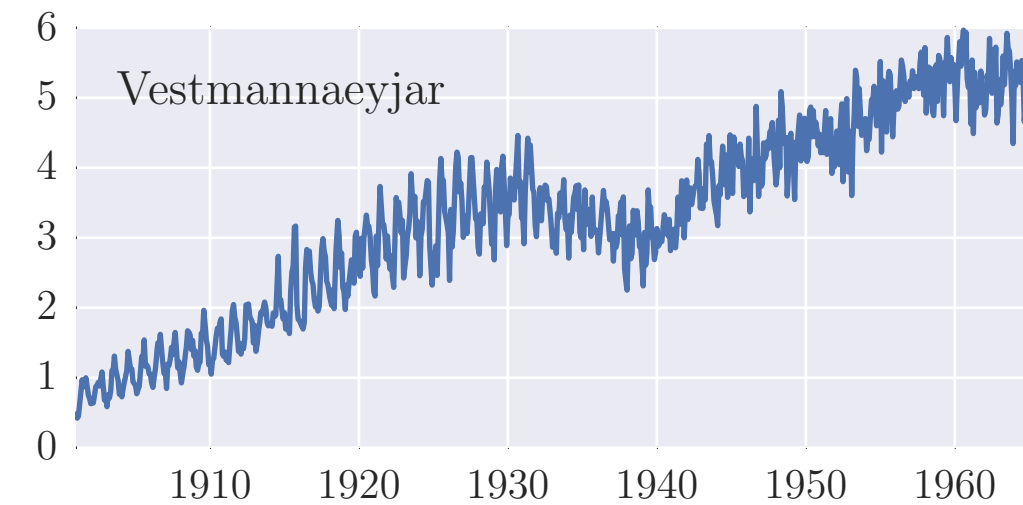

Supplement: Births [file rsif20141125supp2.pdf]
